# Microencapsulation of Pineapple Peel Extract by Spray Drying Using Maltodextrin, Inulin, and Arabic Gum as Wall Matrices

**DOI:** 10.3390/foods9060718

**Published:** 2020-06-02

**Authors:** Sofia C. Lourenço, Margarida Moldão-Martins, Vítor D. Alves

**Affiliations:** LEAF, Linking, Landscape, Environment, Agriculture and Food, Instituto Superior de Agronomia, Universidade de Lisboa, Tapada da Ajuda, 1349-017 Lisboa, Portugal; sofiaclourenco@isa.ulisboa.pt (S.C.L.); mmoldao@isa.ulisboa.pt (M.M.-M.)

**Keywords:** encapsulation, pineapple peel extract, maltodextrin, inulin, arabic gum, spray drying

## Abstract

A pineapple peel hydroalcoholic extract rich in phenolic compounds, was stabilized by microencapsulation using spray drying technology, with maltodextrin, inulin, and arabic gum as wall materials. The influence of the type of wall material and drying temperature (150 and 190 °C) on the particles properties was studied. The particles presented a spherical shape with a diameter ranging from approximately 1.3 to 18.2 µm, the exception being the ones with inulin that showed a large degree of agglomeration. All powders produced presented an intermediate cohesiveness and a fair to good flowability according to Carr index and Hausner ratio, which envisages suitable handling properties at an industrial scale. The microencapsulation processes using maltodextrin and arabic gum at 150 °C were the ones that showed higher maintenance of the antioxidant activity of compounds present in the extract before encapsulation during spray drying. In addition, the microparticles obtained were quite efficient in stabilizing the encapsulated phenolic compounds, as their antioxidant activity did not change significantly during six months of storage at 5 °C.

## 1. Introduction

Pineapple is a tropical fruit widely consumed in many countries due to its exotic aroma and pleasant flavor, in addition to its health-promoting properties. Beyond its consumption as fresh fruit, various processed products are commercially available like fresh-cut and canned products and juices. During pineapple processing, a high amount of byproducts (e.g., peel and core) are generated, which become industrial residues that need to be readily separated from the production line as they are prone to microbial growth. These rejected residues constitute a source of additional costs associated with their disposal. However, they are still rich in natural valuable compounds, namely vitamin C, enzymes, fiber, minerals, and phenolic compounds, which have the potential to be used for functional food ingredients [1,2,3,4]. The pineapple peel was found to have antioxidant and biological activity due to the vitamins, flavonoids such as myricetin, and phenolic acids such as caffeic acid, p-coumaric acid and ferulic acid [3,5]. The intake of plant-derived phenolic compounds supports the antioxidative defense of the human body and may play a vital role in human health through protection against several illnesses, such as cancer, cardiovascular diseases, diabetes, osteoporosis, and neurodegenerative diseases [6,7]. To take advantage of these compounds, the production of bioactive extracts from pineapple peel for future applications is of significant interest. Pineapple peel extracts have been studied, for example, to delay lipid oxidation in food products due to their antioxidant potential [1]. However, the direct incorporation of these extracts into food products may reduce the effectiveness of their components, due to degradation/oxidation during food processing. In addition, their stabilization during the storage period between production and utilization must be ensured, which may be achieved by microencapsulation [8].

Microencapsulation is a methodology widely studied for the stabilization of valuable compounds extracted from natural resources (e.g., agroindustry wastes) such as vitamins, pigments, flavors, and antioxidants, and their release into food products [9,10,11,12,13]. The microencapsulation process consists of the production of structures, in most cases particles, in which the bioactive compound in the solid, liquid, or gas phase (the core material) is entrapped in one or more classes of wall materials that form a protective barrier [8]. These particles may present a wide range of morphologies and sizes. Typically microparticles have diameters from 1 to 1000 µm [14]. There are various encapsulation techniques, however, the most commonly used in the food industry is spray drying [15]. Usually it represents a viable process for microencapsulation of active compounds due to the simple, economical, flexible, reproducible, and continuous operation, with easy scale up, in comparison with other encapsulation processes, such as coacervation, fluid bed coating, spray cooling/spray chilling, and freeze-drying [8,15].

Biopolymers have attracted the attention of the scientific community as wall materials for the microencapsulation of numerous active compounds due to their interesting chemical, physical, and thermal properties. The selection of suitable wall materials that avoid changes in the extract composition is a crucial point for the success of the encapsulation process [16]. Thereby, the literature reports wall materials that are generally recognized as safe, such as inulin [17,18], maltodextrin with different dextrose equivalent (DE) values [19] and arabic gum [18,20], that have been used for bioactive compound stabilization by spray drying. Maltodextrin is a polysaccharide that consists of β-_D_-glucose units linked mainly by glycosidic bonds, obtained by acid or enzyme hydrolysis of some starches (corn, rice, potato, starch, or wheat). Variations in DE values result in powders with different physico-chemical properties, such bulk density, water absorption, water solubility, hygroscopicity, and porosity [21]. Among other characteristics, maltodextrins have high solubility in water, low viscosity even at high solid content, neutral flavor, and colorless solutions, and they are readily available [22,23]. This wall material has been mostly used in products that are difficult to dry, with the purpose of decreasing stickiness and agglomeration problems during storage [24]. Inulin is a reserve polysaccharide produced by many plants and is often extracted industrially from chicory. It is a polymer of fructose units linked with a terminal glucose unit at the end of the chain. It is referred as presenting functional activities, such as improvement of calcium bioavailability, and biological effects like anticytotoxic and immunomodulatory properties [25,26]. In addition, it behaves as a prebiotic, stimulating the activity of colon-beneficial microflora [27]. Since its release takes place only in the intestine, this polymer can be used for the protection of the bioactive compounds susceptible to degradation along the human digestive tract. Arabic gum is a complex heteropolysaccharide with a highly-branched structure, consisting of D-glucuronic acid, L-rhamnose, D-galactose, and L-arabinose, including approximately 2% protein. It has been used as wall material in spray drying due to its good emulsification properties, high solubility, and low viscosity in aqueous solutions. In addition, it provides good retention of volatile substances and confers effective protection against oxidation [22,28]. These three biopolymers were selected to be used in the present work due to their functional properties, in addition to their technological characteristics.

The main objective of this work was the valorization of the phenolic compounds from pineapple peel, going beyond the studies presented to date, regarding their extraction followed by stabilization using the spray drying process. The work focused on studying the influence of spray drying process conditions, in terms of different wall materials (maltodextrin, inulin, and arabic gum) and inlet air drying temperature (150 and 190 °C), on the properties of the particles obtained. It was intended to produce microparticles loaded with encapsulated compounds with antioxidant activity that were stable over long storage periods. In addition, the aim was to obtain stable microparticles with suitable physical properties to be handled on an industrial scale.

## 2. Materials and Methods

### 2.1. Pineapple Peel and Wall Material

Pineapple (*Ananas comosus*) of Sweet Gold variety, imported from Argentine, was purchased at the local market (Lisboa, Portugal, November 2019) and peeled at the laboratory. A mass of 4.9 kg of peel was separated from other edible parts of 12 randomly chosen pineapples. Commercial maltodextrin (Sigma-Aldrich 4-7 DE, St. Louis, MO, USA), inulin (Alfa Aesar 22-25 DP, Haverhill, MA, USA), and arabic gum (Laborspirit Lda., Santo Antão do Tojal, Portugal) were used as wall materials.

### 2.2. Extract Preparation

Pineapple peel was minced using a food processer at 10,000 rpm. The minced peel had a total soluble solids content of 9.27 ± 0.60 ºBrix. Afterwards, a solid–liquid extraction was performed by adding a water:ethanol mixture (20:80 *w*/*w*) as solvent and stirring during 25 min at ambient temperature in the dark. A mass:volume ratio of 1:1 (pineapple peel:solvent) was used. The mixture was subsequently filtered through a cotton fabric filter and the permeate was centrifuged at 7000 rpm for 10 min at 15 °C (HERMLE Labortechnik 383 K, Germany). After centrifugation, the ethanol of the supernatant was evaporated using a rotary evaporator (Rotavapor R II—Buchi, Flawil, Switzerland). The final aqueous solution (extract) was weighed, collected in amber flasks, and stored away from light, until further analysis.

### 2.3. Spray Drying Process

The effect of different wall materials (maltodextrin, inulin, and arabic gum) and inlet air temperature (150 and 190 °C) was evaluated. For the preparation of the feed solution, the wall material was gradually added to the aqueous extract (5% *w*/*w*) obtained in the previous section, under magnetic stirring (ARE, Velp). In the case of inulin, the extract was preheated at 80 °C prior to its addition to enable its fast dissolution. The solutions were fed to a spray dryer model SD-05 (Lab-plant SD-05, Filey, UK), with co-current flow regime, equipped with a 0.5 mm diameter nozzle. The inlet feed flow rate was set at 3.7 mL/min, the pressure of the compressed air was set at 1.7 bar and the drying air flow rate was set at 47 m^3^/h. Feed solutions were dried at an inlet air temperature of 150 and 190 °C. Moreover, microparticles obtained without the extract used as control were produced. The dried powders collected were stored in sealed amber flasks in the dark at 5 °C.

### 2.4. Analysis of Microparticles

#### 2.4.1. Particle Morphology and Size Distribution

The morphology of the particles obtained by spray drying was observed by scanning electron microscopy (SEM), using a FEG-SEM JEOL JSM7001F equipment. The samples were coated with a mixture of gold (80%) and palladium (20%) in a vacuum chamber and analyzed using a Hitachi S2400 scanning microscope operated at 15 kV. The particle size distribution was determined by measuring the diameter of the particles localized in a selected area of SEM images.

#### 2.4.2. Moisture Content

The moisture content was determined by drying the samples at a temperature of 105 °C until constant weight. Three replicates were carried out for each type of particles.

#### 2.4.3. Wettability and Solubility

Wettability was evaluated according to the method described by A-sun et al. [29] with some modifications. It was evaluated as the time(s) needed for a mass of powder (50 mg) placed on the surface of a fixed distilled water volume (20 mL) to completely submerge without agitation. The tests were performed at 20 °C.

The solubility of the powder was carried out according to the Ee et al. [30]. A mass of 100 mg of powder was dispersed in 10 mL of distilled water using a magnetic stirrer (ARE, Velp) for 5 min at 20 °C. The solution was then centrifuged (HERMLE Labortechnik Z 383 K, Wehingen, Germany) at 5100 rpm for 10 min. The supernatant was transferred to a pre-weighed aluminum dish and oven-dried at 70 °C overnight. The solubility of the powder (%) was determined by the ratio between the mass of dried supernatant and the initial mass of powder added.

#### 2.4.4. Bulk Density and Bulk Tapped Density

The bulk density (ρ_b_) and bulk tapped density (ρ_t_) of the powder were measured following the procedure described by Fernandes et al. [22]. A pre-weighed graduated cylinder of known volume (2 mL) was gradually filled with the powder. After weighing the filled cylinder, the bulk density was calculated by the mass/volume ratio (g/mL).

The tapped density was obtained by tapping manually, 5 times, a 5 mL graduated cylinder with approximately 300 mg of powder until an insignificant change in volume between successive measurements was verified. Given the mass (m) and the apparent tapped volume (v) of the powder, the ρ_t_ was expressed as *m*/*v* (g/cm^3^).

#### 2.4.5. Flowability and Cohesiveness

The flowability and cohesiveness of the powder were determined in terms of Carr index (CI) and Hausner ratio (HR) using the bulk and tapped density, according to the following equations [31]:

(1) $$\mathrm{CI}=\frac{\left(\rho t-\;\rho b\right)}{\rho t}\;\times \;100$$

(2) $$\mathrm{HR}=\frac{\rho t}{\rho b}$$

The classification is obtained based on CI and HR values presented in Table 1.

#### 2.4.6. Moisture Adsorption Isotherms

The moisture adsorption isotherms were determined by a gravimetric method, according to Alves, et al. [32]. Duplicate samples of spray dried powders were placed in pre-weighed aluminum dishes, dried in a vacuum chamber at 70 °C overnight (WTB-BINDER, Tuttlinge, Germany) and afterwards stored in a desiccator for 24 h. After this time, the samples were weighed and placed in desiccators with five saturated salt solutions: LiCl, Mg(NO_3_)_2_.6H_2_O, NaCl, KCl, and BaCl_2_ with a water activity at 20 °C of 0.122, 0.547, 0.757, 0.854, and 0.91, respectively [33]. The desiccators were maintained at room temperature and the samples were weighted until constant mass.

Equilibrium moisture content (X, dry basis) was plotted against the corresponding water activity and data were fitted with a nonlinear regression with a 5% significance level to the Guggenheim-Anderson-de Boer (GAB) sorption model:

(3) $$X=\frac{X_{0\;}{C\;K\;a}_{w}}{\left(1-{K\;a}_{w}\right)\left(1-{K\;a}_{w}+{C\;K\;a}_{w}\right)}$$

where X_0_ is the monolayer value (% dry basis), a_w_ the water activity, and C and K, are constants. The goodness of the fit was estimated by the determination of minimum standard error of estimate (SE), minimum root means square error (RMSE), minimum mean absolute percentage error (P), and the maximum R-square (R^2^), calculated as follows:

(4) $$SE=\sqrt{\frac{\sum_{i=1}^{N}{\left(Y-Y_{0}\right)}^{2}}{DF}}$$

(5) $$RMSE=\sqrt{\frac{1}{N}\overset{N}{\underset{i=1}{\sum }}{\left(Y-Y_{0}\right)}^{2}}$$

(6) $$P\;\left(\%\right)=\frac{100}{N}\overset{N}{\underset{i=1}{\sum }}\frac{\left|Y-Y_{0}\right|}{Y}$$

where, *Y* is experimental value of equilibrium moisture content, *Y*_0_ the predicted value by the model, *N* the number of observations, and *DF* represents the degrees of freedom of the regression model.

### 2.5. Evaluation of Particles Loading and Antioxidant Activity of Encapsulated Compounds

#### 2.5.1. Preparation of Extracts from Microparticles

For the quantification of the particles’ total phenolic content and the antioxidant activity of the encapsulated pineapple peel compounds, extracts from microparticles were obtained by the method described by Rocha et al. [34] with some modifications. The spray dried particles (200 mg) were dispersed in 10 mL of methanol and strongly homogenized with an ultraturrax homogenizer (IKA Labortechnik T25 Basic, Staufen im Breisgau, Germany) at 13,500 rpm for 3 min in order to break the particles. The suspension was left at 10 °C, in the dark, for 12 h. Then, the solutions were centrifuged at 7000 rpm, during 15 min at 4 °C and the collected supernatant was stored in amber glass flasks.

#### 2.5.2. Total Phenolic Content (TPC)

TPC was determined by direct measurement of the absorbance of the extracts at 280 nm (UNICAM, UV/Vis Spectrometer—UV4, Waltham, MA, USA), as performed by other authors [35,36,37]. A calibration curve was performed with gallic acid (Sigma Aldrich) in water with concentrations from 0 mg/L to 50 mg/L. The particle loading was expressed as mass of gallic acid equivalents (GAE) per mass of particles (mg GAE/g of particles).

#### 2.5.3. Antioxidant Activity (AOA)

##### DPPH Assay

Antioxidant activity of extracts was analyzed by the scavenging ability of the 1,1–diphenyl-2-picrylhydrazyl (DPPH) radical, as described by Huang et al. [38], with some changes. A stock solution was prepared by dissolving 24 mg of DPPH (Sigma Aldrich) in 100 mL of methanol (Sigma Aldrich). This stock solution was kept in a freezer for at least 2 h before use. A working solution was prepared, in an amber flask, by diluting the stock solution (10 mL) in methanol (45 mL) to have an absorbance below 1.1 measured at 515 nm. An aliquot of 0.1 mL of the extracts was added to 3.9 mL of the working solution in amber flasks and left to react in the dark at ambient temperature after being shaken vigorously. The absorbance of the mixtures was measured at 515 nm after 40 min. A reference solution with 0.1 mL of methanol and 3.9 mL of the working solution was used as a blank sample. The measurements were performed in triplicate. The radical scavenging activity (RSA) was measured by Equation (7).

(7) $$\mathrm{RSA}\;(\%)=\frac{Abs_{blank}-Abs_{sample}}{Abs_{blank}}\times 100$$

RSA values were converted into Trolox concentration using solutions with concentrations ranging from 100 to 2000 µM that were made to react with the DPPH radical in the same conditions as the samples. The antioxidant activity of the samples was expressed as Trolox equivalent antioxidant capacity (TEAC), quantified in this study as µmol of Trolox equivalents per mass of dry particles. In the case of the pineapple peel extract before encapsulation, it was expressed per mass of dry extract.

##### Ferric-Reducing Antioxidant Power (FRAP)

FRAP measures the reduction of a ferric-tripyridyltriazine complex (Fe^3+^–TPTZ) to its ferrous form (Fe^2+^- TPTZ) in the presence of antioxidant components. FRAP assay was carried out using the modified method of Suárez et al. [39]. The working FRAP reagent solution was prepared by mixing 25 mL of the acetate buffer 0.3 M, with 2.5 mL of 2,4,6-Tris(2-pyridyl)-s-triazine (TPTZ) 0.01 M and 2.5 mL of the FeCl_3_.6H_2_O solution 0.02 M. The antioxidant activity of the extracts was measured by mixing 90 µL of sample with 270 µL of deionized water and with 2.7 mL of the working FRAP solution. A reference sample was analyzed by adding 90 µL of deionized water instead of a sample. The reaction was carried out in a water bath at 37 °C for 30 min. After this time, the absorbance was measured at 595 nm. Trolox (100–2000 µM) and ferrous sulfate (500–2000 µM) were used as references. The antioxidant activity of the samples was expressed as TEAC and as ferrous sulfate concentration per mass of dry particles. In the case of the pineapple peel extract before encapsulation, it was expressed per mass of dry extract.

### 2.6. Statistical Analysis

Significant differences were determined by one-way analysis of variance (ANOVA) followed by Tukey’s test. Significant levels were defined with α = 0.05. All statistical analyses were carried out using the data analysis software system STATISTICA TM version 8.0 (StatSoft Inc., Tulsa, OK, USA, 2007).

## 3. Results and Discussion

### 3.1. Morphology and Particle Size Distribution

Particles obtained without extract are white colored while the ones with encapsulated pineapple peel extract present a yellow color, independent of the wall material used.

**Figure 1:**
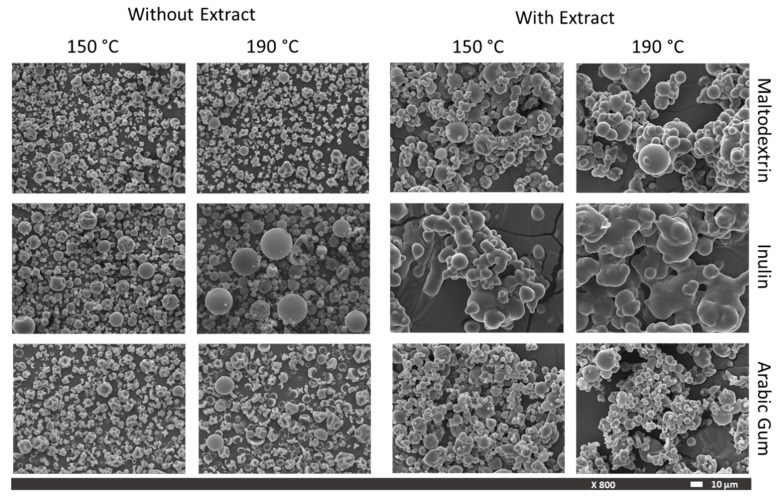
Scanning Electron Microscopy (SEM) images of particles obtained with different wall materials and drying temperatures, with and without extract.

SEM images of the particles with different wall materials and drying temperatures are presented in Figure 1. It can be observed that the particles showed a spherical shape for all feed compositions and drying temperatures. However, the control particles, that are the ones obtained only with the wall material without extract, showed an irregular shape with higher shrinkage and number of concavities on their surface (especially those from maltodextrin and arabic gum), but with a low agglomeration degree. Conversely, the particles with extract showed a smoother surface and higher degree of agglomeration. Similar behavior was observed in particles of orange peel extract encapsulated with whey protein isolate [40], jussara pulp encapsulated with starch, inulin, and maltodextrin [41], and pequi pulp with maltodextrin and arabic gum [42]. These particles presented a spherical morphology after production, but they tended to establish bridges to connect to each other by using the available moisture or absorbing moisture from the environment. In addition, during drying, agglomeration may occur from the collision of particles, which may result in smaller particles co-existing around larger ones. This particles agglomeration may provide higher stability to the microencapsulated compounds, as the outer particles may protect the inner ones [42].

There is evidence from SEM images that all particles obtained are skin-forming structures with a void inner volume (microcapsules). Vacuole formation results from the shrinking process that occurs after the hardening of the outer surface followed by the expansion of the air bubbles trapped inside the droplets, typically during the final stages of the drying process [22]. Besides some broken particles, the large majority do not show relevant evidence of fissures on their surfaces, which is an important fact to ensure the protection of pineapple peel extract from oxidation reactions [41].

When pineapple peel extract was encapsulated with inulin under the different drying conditions, a large difference in the particles’ morphology was observed when compared to that obtained with the other wall materials. The particles’ agglomeration resulted in structures that were far from a spherical shape. This fact may be attributed to specific wall material–extract interactions that may take place when inulin is used. A change in capsule structure was also observed for inulin capsules with encapsulated oregano essential oil [25].

**Figure 2:**
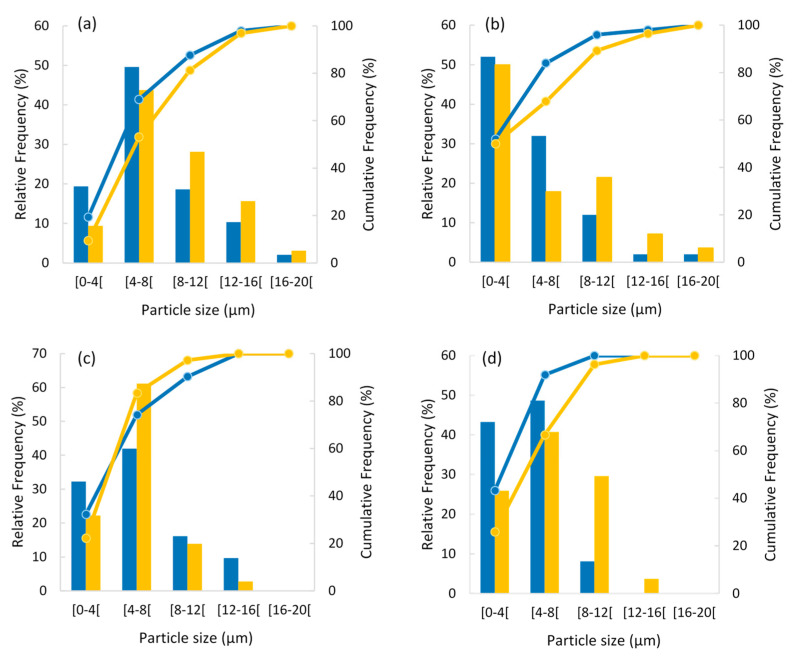
Relative frequency (bars) and cumulative frequency (lines) of the particles’ diameter: (**a**) maltodextrin with extract, (**b**) arabic gum with extract, (**c**) maltodextrin without extract, and (**d**) arabic gum without extract, dried with an inlet air temperature of 150 °C (blue color) and 190 °C (yellow color).

According to the literature, the diameter of the spray dried particles depends on the atomization method used, the properties of the materials, the concentration and viscosity of the feed solution, and drying conditions [22]. The particles’ size influences their industrial application since it has an impact on the powders’ flowing, rehydration capacity, solubility, and density [43]. In this work, the size of inulin particles was not evaluated due to their large aggregation when containing pineapple peel extract. For the other materials, control particles and particles with pineapple peel extract displayed a size distribution from approximately 1.3 to 18.2 µm, and between 81% and 100% of the particles produced showed a diameter lower than 12 µm, depending on the process conditions (Figure 2).

In general, the presence of bioactive compounds led to a decrease in the particle size, since the pineapple-peel-loaded particles had mean diameters of up to 18.2 µm, while particles without extract presented a diameter ranging between 2 to 12.5 µm (Figure 2c,d). This behavior was similar to that reported by Lourenço et al. [44] for FucoPol skin-forming particles, where the average diameter decreased upon encapsulation of bioactives. However, in the case of arabic gum, the fraction of particles with a size between 4 and 8 µm increased, with a decrease in the fraction of the lower diameter particles. In addition, powders obtained with the lower drying temperature tested (T = 150 °C) tended to present a higher percentage of smaller particles.

### 3.2. Powder Properties

The physical properties of powders constituted by microparticles with encapsulated pineapple peel extract produced at different inlet air temperature with three wall materials are presented in Table 2.

#### 3.2.1. Moisture Content

The moisture content of the particles in the present study ranged from 1.87% to 6.29%, close to the values reported in other studies using different wall materials and similar spray drying conditions, such as pineapple juice with maltodextrin, cactus pear extracts with maltodextrin and inulin, and acerola pomace extract with maltodextrin and cashew tree gum [45,46,47].

The data obtained in this study showed that an increase in inlet air temperature does not lead to a significant change in the moisture content of the inulin particles. However, the samples prepared with arabic gum and maltodextrin at 150 °C drying temperature had a significantly higher moisture content compared to those obtained at 190 °C. An increase in inlet air temperature led to a significant moisture reduction of these powders. This may have occurred as a result of different drying rates between the feed solution droplets and drying air due to their different physical properties. In addition, the different chemical properties of the wall materials will induce different residual moisture contents for each drying temperature. According to TA Tran et al. [48], arabic gum was assumed to have an ability to absorb water from surrounding environments to a higher extent than maltodextrin. Observing all the wall materials, the arabic gum particles dried at 150 °C are the ones with the highest moisture content. On the other hand, when dried at 190 °C, arabic gum particles are the ones with the lowest moisture content.

#### 3.2.2. Wettability and Solubility

The behavior that powders may present in food formulations can be evaluated from different proprieties of reconstitution. This phenomenon is characterized by the wetting, dispersing, sinking, and dissolving action of powder particles [49]. According to Seth et al. [50], wettability is the ability of the powder to absorb liquid water. The present results showed a wettability time of powders varying from 82 to 305 s. The highest values were found for powders produced with maltodextrin. In addition, an increase in wettability time values of maltodextrin powders was perceived as the inlet air temperature decreased. The same behavior was observed for arabic gum powders. This fact may be due to the higher percentage of lower size particles produced at lower temperature, leading to lower void spaces between particles, increasing the resistance to water transfer by capillarity to the floating powders.

The solubility value is an important property that can potentially affect the availability of the encapsulated compounds when loaded particles are incorporated in a food system [18]. In addition, poorly-soluble powders can cause processing difficulties. Table 2 shows that solubility values ranged from 62% to 75%, which revealed that all powders are relatively soluble in water. These values were expected since these materials are mostly used in spray drying processes due to their high solubility. Similar results have been reported for spray dried grape skin powders with added arabic gum, polydextrose, and partially hydrolyzed guar gum [51].

The wettability and solubility depends on the surface area and particle size, and the presence of amphipathic substances and their chemical structure. According to the SEM analysis, when the inlet temperature increased for the same wall material, powders with larger particles size were produced. These tended to have higher void volumes and be heavier particles, which may have facilitated their immersion, whereas powders with smaller particles tend to float on the water’s surface. Thus, powders with smaller particles, produced at low inlet air temperature, presented a lower wettability (more time at water surface) and lower solubility, as shown in Table 2.

#### 3.2.3. Bulk Density, Bulk Tapped Density, Flowability, and Cohesiveness

The bulk properties are important factors for transport and packaging, as these variables are useful for determining the amount of material that will fit inside a container [22]. High bulk density results in higher packing due to the lower volume occupied per mass unit. Lower bulk density values may increase the possibility of product oxidation, due to the large amount of air within the powder, reducing storage stability. The bulk density values of the samples ranged from 0.18 to 0.30 g/cm^3^. Values close to these were obtained for encapsulated rosemary essential oil in arabic gum, starch, maltodextrin, and inulin wall materials (0.23–0.35 g/mL) [22].

Particle size distribution is a factor that may affect bulk density. It is expected that lower-particle-size powders present a higher bulk density. In this work, the particle size tended to be lower when decreasing the drying temperature for arabic gum and maltodextrin wall materials (Figure 2a,b). However, a higher bulk density was observed for arabic gum powders obtained at lower temperature, but not for the maltodextrin ones. The correlation between particles size and bulk density is not linear, probably because the particles formed are hollow with a thin wall, as observed on SEM images (Figure 1). Spray dryer inlet temperature had no significant effect (*p* < 0.05) on the bulk tapped density that ranged from 0.24 to 0.40 g/cm^3^.

In relation to handling properties, lower Hausner ratio (HR) values indicate desirable cohesiveness properties that are correlated to better flowability characteristics. A free-flowing material has a low tendency for further consolidation, which is helpful for preventing production stoppages at industrial scale. The powders in the present study did not present significant statistical differences (*p* < 0.05) regarding flowability and cohesiveness. These results are in line with the bulk tapped density values. Generally, the higher the cohesiveness, the larger the powder collapse observed on tapping [52]. According to Table 1, this may be attributed an intermediate cohesiveness and a fair to good flowability for all powders. Still, it may be a tendency for the inulin powders dried at 190 °C to present better flowing properties than the others.

### 3.3. Water Vapour Adsorption Isotherms

**Figure 3:**
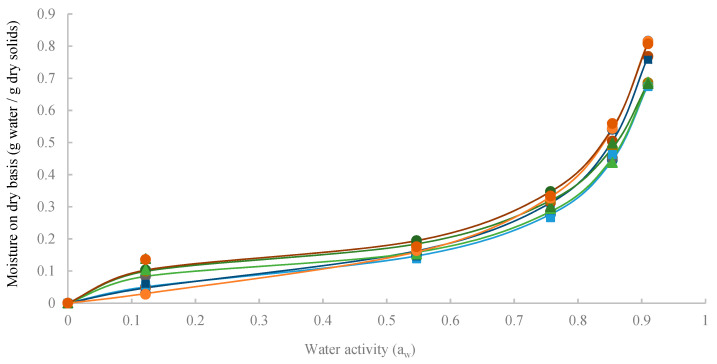
Water adsorption isotherms of particles obtained at 20 °C. Guggenheim-Anderson-de Boer (GAB) equation (lines). Experimental data: maltodextrin at 150 °C (■), maltodextrin at 190 °C (■), inulin at 150 °C (▲), inulin at 190 °C (▲), arabic gum at 150 °C (●), and arabic gum at 190 °C (●).

The knowledge of water vapour adsorption capacity is important in powder processing, as it influences its physical properties, enabling for example prediction of the behavior upon mixing, moisture changes during storage, and shelf-life stability [24]. In order to evaluate the vapour adsorption of powders with encapsulated extract, they were stored under different relative humidity conditions, and water adsorption isotherms were assessed at 20 °C (Figure 3). It can be seen that all the curves’ behavior was of type II, the nonlinear sigmoidal or S-shaped adsorption isotherm, according to Brunauer’s classification [53].

The adsorption isotherms exhibited an increase in equilibrium moisture content with an increase in water activity for all the particles studied, which is characteristic of amorphous materials rich in hydrophilic components. This behavior can be attributed to the hydrophilic nature of carbohydrates and protein present in spray dried pineapple powder.

It is possible to define two main zones, a multilayer adsorption region where moisture content increased linearly at low and intermediate water activities, and a capillary condensation region, where water content rapidly increased with water activity at high water activity values. This trend has been reported in the literature for many powder materials such as tomato pulp, orange juice, and tamarind pulp powders [54,55,56].

Experimental data (Figure 3) indicates that the variation in the equilibrium moisture content with water activity was quite similar for all powders tested. In most cases, there was not a substantial effect of the type of wall material and drying inlet air temperature on the water vapor adsorption capacity. Still, for the case of arabic gum particles dried at 150 °C, this powder was the least hygroscopic of all products at low water activity (a_w_ = 0.12), changing its behavior, becoming the one with higher water content at high water activity (a_w_ = 0.91). Also, the lowest level of water adsorption under conditions of high relative humidity (a_w_ = 0.91) was observed for maltodextrin powder dried at 150 °C.

The GAB model was successfully fitted to experimental data by non-linear regression analysis and the estimated values of the GAB coefficients are presented in Table 3. The parameter C determines the strength of the water primary binding sites on the surface of the powder. The higher the C value, the more binding sites are present. The value of K provides a measure of interactions between the molecules in multilayers with the powder and tends to fall between the energy value of the molecules in the monolayer and that of liquid water. If K is equal to 1, the multilayers have the same characteristics as liquid water. M_0_ indicates the amount of water that is adsorbed in a monolayer on the surface of the powders and is a measure of the availability of active adsorption sites [24].

According to Lewicki [57], in order to describe a good sigmoid type of adsorption isotherm, the parameters of the GAB equations should be in the following ranges: C ≥ 5.67 and 0.24 < K ≤ 1. The results obtained in the present work (Table 3) are in accordance, the exception being the arabic gum particles dried at 150 °C.

The estimated monolayer moisture content (M_0_) for all powders was between 7.31% and 9.94% (dry basis). Though the comparison of data with the literature is difficult, since the materials used and their concentration was not the same, it is worth mentioning that M_0_ values were similar to those reported for pineapple pulp powder with 18% of maltodextrin concentration (M_0_ from 5.8% to 6.9% dry basis) and with 18% of arabic gum (M_0_ from 6.7% to 7.9% dry basis) at a temperature in the range of 20–50 °C [24]. The parameter K was practically not affected by the wall material or inlet air temperature used.

### 3.4. Total Phenolic Content and Antioxidant Activity

**Figure 4:**
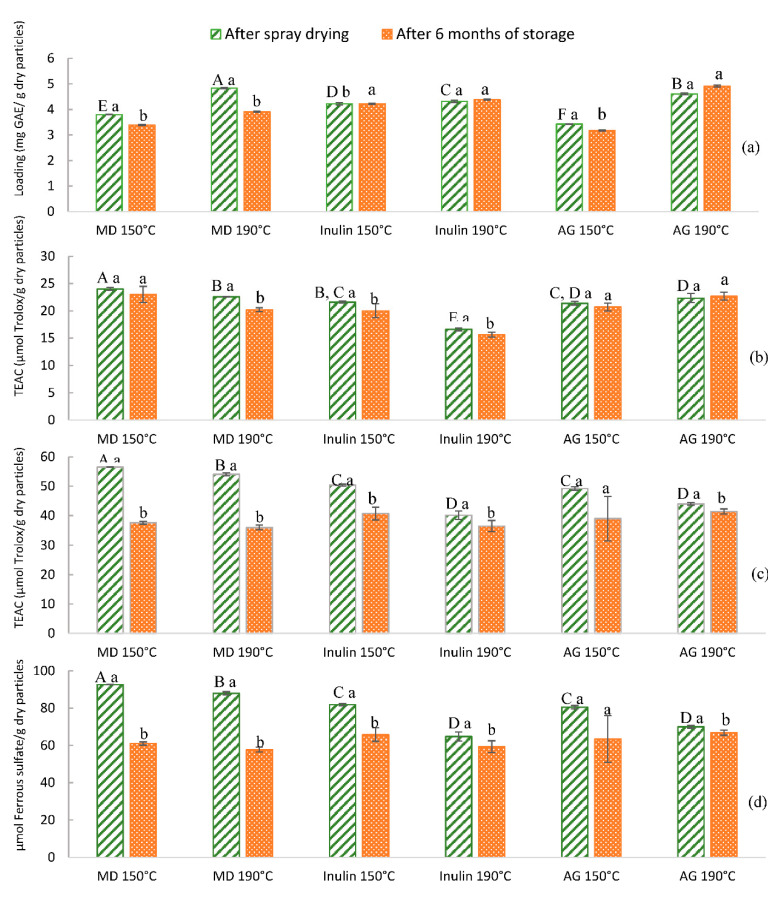
(**a**) Total phenol content of the microparticles produced, (**b**) antioxidant activity (AOA) of the encapsulated extract after spray drying by the 1,1–diphenyl-2-picrylhydrazyl (DPPH) method, (**c**) AOA by the ferric-reducing antioxidant power (FRAP) method expressed as Trolox equivalent antioxidant capacity (TEAC), and (**d**) AOA by the FRAP method expressed as µmol ferrous sulfate. Values are the mean of three different replicates. Different lower-case letters in the same double column show statistically significant differences among values for the same wall material and drying temperature (*p* < 0.05). Different upper-case letters show statistically significant differences among values after spray drying (*p* < 0.05). MD, maltodextrin and AG, arabic gum.

The total phenolic content (TPC) of the extract before encapsulation was 29.33 mg GAE/g dry extract and the respective antioxidant activity (AOA) was 123.64 µmol Trolox/g dry extract with the DPPH method, 281.63 µmol Trolox/g dry extract and 457.24 µmol sulfate ferrous/g dry extract with the FRAP method. Regarding the TPC content of particles with encapsulated extract, it is presented in Figure 4, along with the AOA of the encapsulated compounds right after particle production and after six months of storage at 6 °C in the dark.

The effect of the spray drying process conditions on particles’ TPC is shown in Figure 4a. The TPC expressed as mg GAE/mg dry particles ranged from 3.42 to 4.82, with the highest value observed for maltodextrin particles obtained at 190 °C followed by the ones for arabic gum produced at the same drying temperature. In addition, the wall materials and spray drying conditions used in this study resulted in a small loss of phenolic content during six months of storage at 6 °C, which indicates that all wall materials were effective as protective barriers. The higher loss was observed for maltodextrin particles obtained at 190 °C (around 20%). This fact is in agreement with the reports of other researchers who evaluated the effect of microencapsulation on phenolic compounds. Çam et al. [58] observed a total phenolic content retention, at 4 °C for 3 months, in microparticles obtained by spray drying of pomegranate peel extract with maltodextrin.

The AOA values of encapsulated pineapple peel compounds ranged from 16.6 to 24.1 µmol Trolox/mg of dry particles with the DPPH method, 39.7 to 56.5 µmol Trolox/mg dry particles and 63.9 to 92.6 µmol ferrous sulfate/mg dry particles with the FRAP method (Figure 4b–d). These values of AOA expressed in dry particles basis, corresponded to the following expressed in GAE basis: from 3.8 to 6.3 µmol Trolox/mg GAE with the DPPH method, 8.6 to 14.9 µmol Trolox/mg GAE and 13.9 to 24.4 µmol ferrous sulfate /mg GAE with the FRAP method, respectively. The results show that the loaded microparticles retained most of the AOA of the extract before encapsulation (expressed in GAE basis as 4.22 µmol Trolox/mg GAE with the DPPH method, 9.60 µmol Trolox/mg GAE, and 15.6 µmol sulfate ferrous/mg GAE with the FRAP method) after the spray drying process, regardless of the wall material and drying temperature used.

According to the results, the increase of inlet air temperature from 150 °C to 190 °C led to a decreased AOA, measured with the two methodologies used, for all the wall materials studied. At higher temperatures, the components of pineapple peel which are responsible for antioxidant activity are more easily oxidized [59]. In addition, after particle storage for six months without light and at refrigerated conditions, the AOA of some powders decreased significantly (*p* < 0.05) compared to the AOA results obtained right after encapsulation. Only the AOA, measured with the DPPH method, of maltodextrin particles obtained at 150 °C and of arabic gum particles dried at 150 °C and 190 °C, as well as the AOA measured with the FRAP method of arabic gum particles dried at 150 °C, were shown to be fully preserved.

From the results obtained, the encapsulation conditions using maltodextrin and arabic gum at 150 °C, may be selected as the most adequate, since in addition to retaining the AOA (using DPPH methodology) of the original extract, it did not change significantly after six months of storage. Furthermore, arabic gum powders dried at 150 °C presented a good AOA retention measured with both methodologies, envisaging a high stability of a broader range of antioxidant compounds during storage.

## 4. Conclusions

Maltodextrin, inulin, and arabic gum were studied as wall materials to encapsulate an hydroalcoholic pineapple peel extract by spray drying, and the powders obtained were characterized in terms of physical properties, loading of phenolic compounds, and antioxidant capacity. The wall materials and drying temperatures tested enabled powders with intermediate cohesiveness and fair to good flowability to be obtained, indicating suitable handling properties at an industrial scale. Although particles with encapsulated pineapple peel extract showed differences in their morphology when the wall material was changed, the antioxidant activity of the extract was maintained after encapsulation in all cases. From the results obtained, the encapsulation conditions using maltodextrin and arabic gum at a drying temperature of 150 °C, may be envisaged to be the ones with more potential to produce bioactive powders for the development of food products with improved functional properties. Beyond adequate physical properties and solubility in water, these conditions enabled the antioxidant activity of the original extract after encapsulation to be maintained and this did not change significantly after six months of storage. It is envisaged the application of these microcapsules in the production of active edible films and coatings for use as oxygen barriers (e.g., for meat products and nuts). In addition, they have potential to be used in meat and dairy products, as well in fruit and vegetable juices and pulps.

## Figures and Tables

**Table 1:** Carr index (CI) and Hausner ratio (HR) of powders [31].

CI (%)	Flowability	HR	Cohesiveness
<15	Very good	<1.2	Low
15–20	Good	1.2–1.4	Intermediate
20–35	Fair	>1.4	High
35–45	Bad		
>45	Very bad		

**Table 2:** Properties of powders with encapsulated pineapple peel extract produced with different wall materials and spray drying temperature conditions: MD, maltodextrin; I, Inulin; and AG, arabic gum. Different lower-case letters in the same column show statistically significant differences among values (*p* < 0.05).

Wall Material and Spray Drying Temperature	Moisture Content (%)	Wettability (s)	Solubility (%)	Bulk Density (g/cm^3^)	Bulked Tapped Density (g/cm^3^)	Carr Index (%)	Hausner Ratio
MD 150 °C	4.93 ± 0.36 ^b^	305 ± 2.83 ^a^	68.15 ± 0.78 ^a,b^	0.22 ± 0.01 ^c,d^	0.29 ± 0.02 ^a,b^	24.28 ± 3.75 ^a^	1.32 ± 0.07 ^a^
MD 190 °C	4.36 ± 0.37 ^b^	252 ± 3.54 ^b^	71.00 ± 1.41 ^a,b^	0.27 ± 0.01 ^a^	0.40 ± 0.04 ^a^	32.24 ± 3.57 ^a^	1.48 ± 0.08 ^a^
I 150 °C	2.57 ± 0.41 ^c^	115 ± 6.36 ^c^	64.50 ± 0.71 ^b^	0.24 ± 0.01 ^b,c^	0.32 ± 0.02 ^a,b^	24.43 ± 9.50 ^a^	1.34 ± 0.19 ^a^
I 190 °C	2.42 ± 0.36 ^c^	151 ± 1.41 ^e^	64.00 ± 4.24 ^b^	0.30 ± 0.04 ^a^	0.35 ± 0.03 ^a,b^	13.77 ± 8.21 ^a^	1.16 ± 0.11 ^a^
AG 150 °C	6.29 ± 0.48 ^a^	167 ± 6.36 ^e^	62.00 ± 1.41 ^c^	0.28 ± 0.01 ^a^	0.38 ± 0.07 ^a,b^	24.47 ± 13.73 ^a^	1.35 ± 0.24 ^a^
AG 190 °C	1.87 ± 0.55 ^c^	82 ± 9.19 ^d^	75.00 ± 4.24 ^a^	0.18 ± 0.02 ^d^	0.24 ± 0.01 ^b^	23.18 ± 2.74 ^a^	1.30 ± 0.05 ^a^

**Table 3:** Estimated GAB parameters for particles from pineapple peel extract encapsulated with maltodextrin (MD), inulin (I), and arabic gum (AG) as wall materials. Model constants (C and K), monolayer moisture content (M_0_), standard error of estimate (SE), root means square error (RMSE), and mean absolute percentage error (P).

		Model Constants			Goodness of Fit Parameters			
Wall Material	Temperature	C	K	M_0_	SE	RMSE	P (%)	R^2^
MD	150 °C	11.451	0.9818	0.0731	0.0104	0.0095	3.9343	0.998
MD	190 °C	7.2135	0.9799	0.0845	0.0211	0.0192	8.0371	0.994
I	150 °C	11.451	0.9818	0.0734	0.0125	0.0114	5.1616	0.997
I	190 °C	11.450	0.9585	0.0876	0.0220	0.0201	8.0678	0.992
AG	150 °C	2.6824	0.9712	0.0994	0.0027	0.0027	2.1924	0.999
AG	190 °C	11.4509	0.9760	0.0906	0.0193	0.0176	7.0451	0.995
